# Hypercortisolism Due to Paraganglioma Secreting Adrenocorticotropin and Catecholamines

**DOI:** 10.1210/jcemcr/luaf052

**Published:** 2025-04-10

**Authors:** Drew W Cox, Aleona C Zuzek, Matthew Genco, Tammy Holm, Shailendra B Patel

**Affiliations:** Department of Medicine, University of Cincinnati College of Medicine, Cincinnati, OH 45219, USA; Division of Endocrinology, Diabetes and Metabolism, University of Cincinnati Medical Center, Cincinnati, OH 45219, USA; Division of Endocrinology, Cincinnati VA Medical Center, Cincinnati, OH 45220, USA; Division of Surgical Oncology, University of Cincinnati Medical Center, Cincinnati, OH 45219, USA; Division of Endocrinology, Diabetes and Metabolism, University of Cincinnati Medical Center, Cincinnati, OH 45219, USA

**Keywords:** paraganglioma, ectopic ACTH, Cushing syndrome, hypercortisolism

## Abstract

A paraganglioma is a neuroendocrine tumor classically associated with catecholamine production. We describe a 71-year-old woman with an incidentally identified para-aortic mass who later developed hyperglycemia, hypertension, hypokalemia, and leukocytosis. Work-up ultimately revealed significantly elevated adrenocorticotropin (ACTH), cortisol, and metanephrines, and biopsy of the mass suggested paraganglioma cosecretion of both ACTH and catecholamines. Using osilodrostat to decrease her excess cortisol production, she underwent successful surgical paraganglioma resection. Pathology of the mass demonstrated a paraganglioma with ACTH-producing cells, confirming the diagnosis of ectopic Cushing syndrome (CS). Following resection, the patient had resolution of hypertension and hyperglycemia and normalization of the hypothalamic-pituitary-adrenal axis. We describe the work-up and important perioperative and long-term management considerations for patients with hypercortisolism from ectopic CS and catecholamine excess.

## Introduction

While a paraganglioma is a neuroendocrine tumor classically associated with catecholamine production, these tumors can rarely secrete alternative hormones [1, 2]. We describe the work-up, perioperative management, and outcomes of a patient with a paraganglioma found to express both adrenocorticotropin (ACTH) and catecholamines. Predominant ACTH overproduction drove clinical presentation. Extrapolated from its efficacy in managing Cushing disease (CD), we demonstrate an example of using osilodrostat to manage severe hypercortisolism secondary to peripheral ACTH hypersecretion. We also discuss the challenge of treating hypertension in the setting of concurrent cortisol and catecholamine excess and highlight considerations for prophylaxis against thromboembolism and infection in severe hypercortisolism.

## Case Presentation

A 71-year-old woman with history of invasive ductal cell carcinoma of the breast treated with partial mastectomy and exemestane, primary hypothyroidism, and multiple sclerosis (complicated by neurogenic bowel, neurogenic bladder with chronic Foley, and paraplegia with wheelchair use) presented to the emergency department in July 2023 with 2 days of generalized abdominal pain, nausea, vomiting, and fatigue. She had intermittently elevated blood pressures during prior oncology clinic visits but no formal hypertension diagnosis. A computed tomography (CT) scan of the abdomen and pelvis 1 year earlier incidentally noted a 3.3-cm aortocaval soft tissue mass. Retrospective imaging review demonstrated presence of this mass unchanged in size from 2020. To evaluate for metastatic disease related to her breast cancer history, she had a fluorodeoxyglucose–positron emission tomography (FDG-PET) scan performed in January 2023 that demonstrated heterogeneous increased FDG uptake of the mass, concerning for malignancy or metastasis. As the mass size remained unchanged, the plan was to repeat imaging in 6 months.

## Diagnostic Assessment

**Figure 1.**
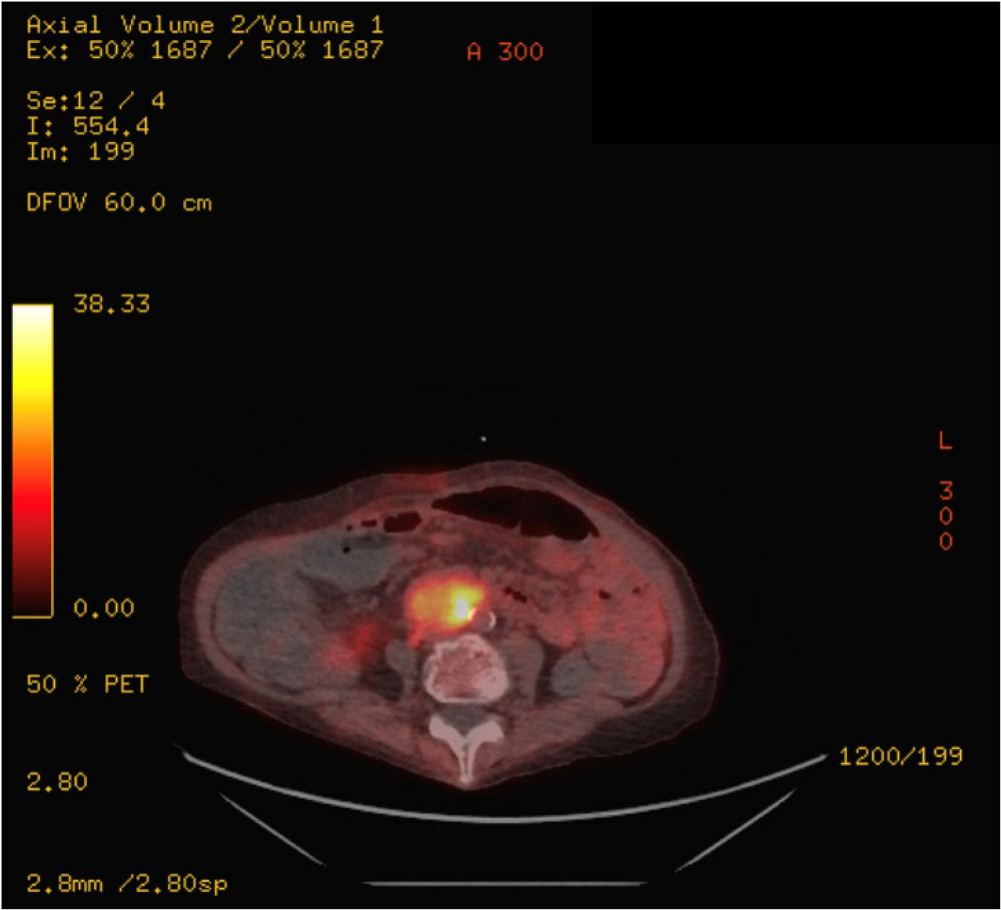
Fluorodeoxyglucose–positron emission tomography (FDG-PET) scan imaging of para-aortic mass. PET imaging showing increased FDG uptake by the para-aortic mass, ultimately found to be a paraganglioma.

Vital signs in the emergency department were notable for blood pressure 155/92 mm Hg and heart rate 114 beats per minute (bpm). Examination generally reassuring with a nontender and soft abdomen. Initial laboratory values were notable for sodium 131 mEq/L (131 mmol/L) (reference range, 136-145 mEq/L, 136-145 mmol/L), potassium 3.0 mEq/L (3.0 mmol/L) (reference range, 3.4-5.1 mEq/L, 3.4-5.1 mmol/L), and white blood cell count 21.9 × 1000/µL (21.9 × 10^9^/L) (reference range, 4.7-11 × 1000/µL, 4.7-11 × 10^9^/L). Admission CT of the abdomen and pelvis demonstrated gastric distension, large stool burden, the known abdominal mass, and a newly nodular left adrenal gland. During admission, systolic blood pressure was frequently 160 to 190 mm Hg and heart rate remained greater than 90 bpm. Blood pressure control remained challenging even with uptitrated losartan 50 mg twice daily and amlodipine 5 mg daily. She had persistent hypokalemia and hypomagnesemia requiring scheduled supplementation, and blood glucoses were as elevated as 349 mg/dL (19.37 mmol/L) (reference range, 80-125 mg/dL, 4.44-6.94 mmol/L) with glycated hemoglobin A_1c_ 6.7% (reference range, ≤6.5%). Leukocytosis peaked at 34.7 × 1000/µL (34.7 × 10^9^/L) with an unrevealing infectious work-up. Her previously ordered follow-up PET scan was obtained inpatient and showed increased FDG uptake of the bilateral adrenal glands and para-aortic mass (now increased in size to 4.2 cm) (Fig. 1). A cortisol obtained at 6 Am was elevated at 100 µg/dL (2759 nmol/L) (reference range, 6.7-22.6 µg/dL, 184.9-623.8 nmol/L), prompting an endocrinology consult. Endocrinology confirmed absence of cushingoid features, but repeat morning cortisol revealed persistent hypercortisolism at 107.91 µg/dL (2977 nmol/L) with concurrently elevated ACTH at 365 pg/mL (80.3 pmol/L) (reference range, 6-50 pg/mL, 1.3-11.0 pmol/L). Twenty-four–hour urine cortisol was significantly abnormal at 8275.6 µg/24 h (22840 nmol/24 h) (reference range, 4.0-50.0 µg/24 h, 11.0-138.0 nmol/24 h). Serum free metanephrines and normetanephrines were respectively elevated to 168 pg/mL (852 pmol/L) (reference range, <58 pg/mL, <294 pmol/L) and 574 pg/mL (3134 pmol/L) (reference range, <149 pg/mL, <814 pmol/L). Other laboratory work included dehydroepiandrosterone sulfate (DHEA-S) elevation to 449 µg/dL (12.17 µmol/L) (reference range, 4-157 µg/dL, 0.11-4.25 µmol/L) and chromogranin A elevation to 1220 ng/mL (1220 µg/L) (reference range, <311 ng/mL, <311 µg/L). Pituitary magnetic resonance imaging noted normal pituitary anatomy. Her morning cortisol, following a supervised overnight dose of 1 mg dexamethasone, was 117 µg/dL (3228 nmol/L) (reference range, <6.7 µg/dL, <185 nmol/L), so ectopic ACTH from the abdominal mass was suspected. Prior to the resulting of metanephrine testing (which may have precluded a biopsy), she underwent endoscopic ultrasound-guided biopsy of the para-aortic mass and left adrenal nodularity to rule out metastatic disease. Pathology of the para-aortic mass aspirate showed clustered tumor cells with neuroendocrine appearance concerning for paraganglioma. ACTH immunostaining was patchy, weak, and nonspecific. The aspirated adrenal nodularity showed typical adrenal tissue. She was discharged with close endocrinology follow-up.

## Treatment

**Figure 2.**
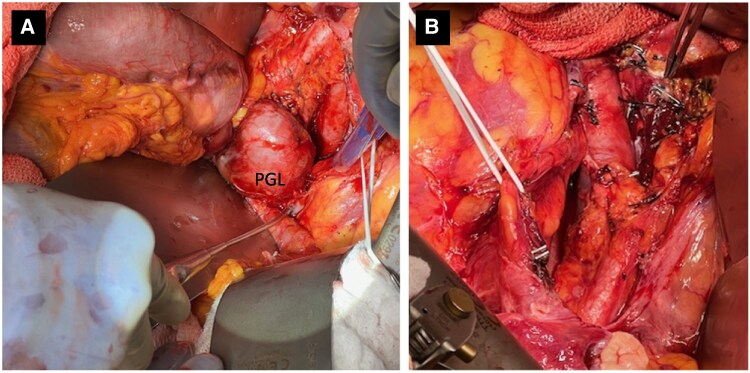
Intraoperative image of paraganglioma pre resection and post resection. A, Intraoperative image of the exposed paraganglioma labeled “PGL.” B, Image of the surgical site status post paraganglioma resection.

A combination of doxazosin 1 mg at bedtime, amlodipine 10 mg daily, and losartan 50 mg daily achieved improved blood pressure control. For the patient’s severe hypercortisolism, she was started on osilodrostat 2 mg twice daily. Urinary tract infection prophylaxis was transitioned to sulfamethoxazole/trimethoprim 400 mg/80 mg daily for concurrent *Pneumocystis jirovecii* pneumonia (PCP) prophylaxis, and she started fondaparinux 2.5 mg daily for thrombosis prophylaxis (prior heparin-induced thrombocytopenia). Paraganglioma resection was recommended once medically stable given the degree of hypercortisolism. Hypertension regimen was adjusted to prioritize α blockade, achieving excellent control on doxazosin 2 mg at bedtime and labetalol 100 mg twice daily. After 2 weeks on osilodrostat, there was a notable reduction in morning cortisol to 62.85 µg/dL (1734 nmol/L) and 24-hour urine cortisol to 2680 µg/24 h (7397 nmol/24 h). Osilodrostat was gently uptitrated to 4 mg twice daily, reaching a nadir morning serum cortisol of 4.27 µg/dL (117.8 nmol/L) and 24-hour urine cortisol of 8.8 µg/24 h (24.29 nmol/24 h). Eight days prior to surgery, she developed symptoms of adrenal insufficiency. Osilodrostat was decreased to 3 mg twice daily and hydrocortisone 20 mg/10 mg twice daily was added for replacement. Preoperatively, she received a stress dose of 100 mg intravenous (IV) hydrocortisone. She underwent paraganglioma resection with surgical oncology without intraoperative complication (Fig. 2). Postoperatively, osilodrostat, doxazosin, and labetalol were discontinued; insulin was dose-reduced; and a steroid taper was started with 50 mg hydrocortisone IV every 6 hours. Seventeen days post operation, she was discharged on twice-daily hydrocortisone 25 mg/10 mg, minimal insulin, and no antihypertensive medications. She started apixaban 5 mg twice daily for provoked postoperative pulmonary embolism.

## Outcome and Follow-up

**Figure 3.**
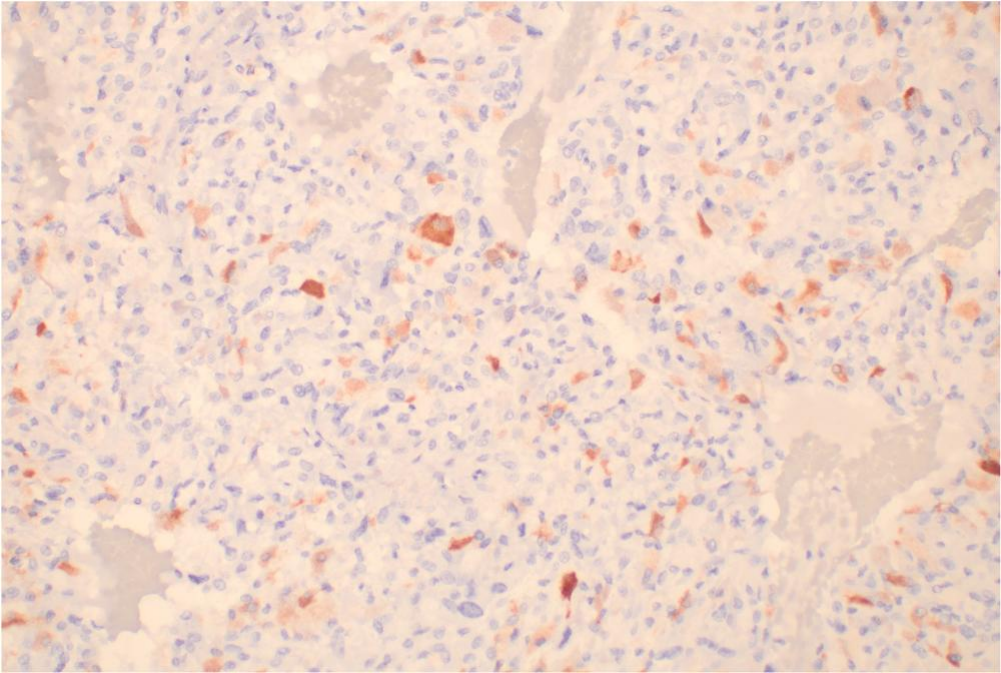
Histopathology of resected paraganglioma. Adrenocorticotropin (ACTH) immunohistochemical stain of tissue from the resected paraganglioma. ACTH-producing cells are stained reddish-brown.

Pathology of the resected mass revealed a 4.6 × 4.3 × 3.3 cm, well-encapsulated, heterogeneous paraganglioma with friable necrotic-appearing and focal hemorrhagic areas. Seven of 7 resected lymph nodes were negative for malignancy. The first ACTH immunohistochemical stain was negative. Due to high suspicion for ACTH secretion, the sample was sent to a second facility that used an alternative antibody, ultimately confirming ACTH positivity in scattered cells (Fig. 3).

**Figure 4.**
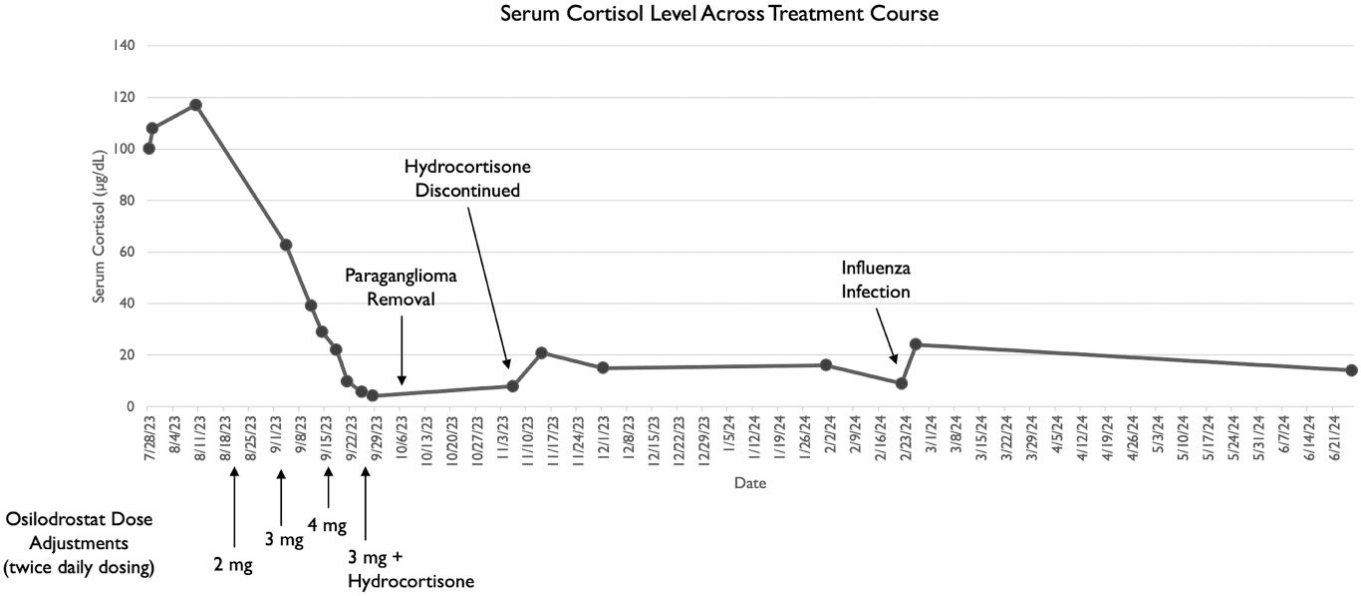
Serum cortisol level across treatment course. Graphing of the patient's serum cortisol levels throughout her treatment course. Each bullet along the graph reflects a serum-measured cortisol level. Key points of medical or procedural management are indicated with an arrow at the time of intervention. Below the x-axis, points of dose adjustment of osilodrostat are indicated.

At an appointment 19 days after surgery, the patient reported symptomatic improvement. Hydrocortisone was fully tapered off on postoperative day 39 with a reassuring morning ACTH of 48 pg/mL (10.57 pmol/L) and cortisol of 20.8 µg/dL (573.8 nmol/L) (Fig. 4). PCP prophylaxis was discontinued. Repeat abdominal CT 4 months post surgery showed no mass recurrence. Laboratory values at this time remained stable with random ACTH 33 pg/mL (7.28 pmol/L), cortisol 16.05 µg/dL (442.8 nmol/L), chromogranin A 257 ng/mL (257 µg/L), and 24-hour urine cortisol 27.5 µg/24 h (75.9 nmol/24 h). Five months postoperatively, blood pressure and glucose remained controlled without medication. Laboratory values at this time showed a mild increase in plasma free metanephrines to 59 pg/mL (299 pmol/L) and cortisol to 24.22 µg/dL (668.1 nmol/L) attributed to influenza infection. Testing for genetic associations with paraganglioma was negative and repeat CT remained negative for recurrence. Surveillance laboratory values were initially monitored every 12 weeks.

## Discussion

A paraganglioma is a neuroendocrine tumor that arises from paraganglia and can be categorized as sympathetic or parasympathetic depending on cell of origin [2]. Classic sympathetic paragangliomas solely secrete catecholamines, though most remain clinically silent. This case is unique as the paraganglioma cosecreted ACTH, resulting in severe hypercortisolism. Delineating the primary driver for symptoms is challenging as cortisol and catecholamine excess can have overlapping symptoms. However, the degree of hypercortisolism in this patient suggests cortisol as the primary driver. Similar cases of ACTH-secreting paragangliomas have been reported but are rare [1]. Upward of 40% of paragangliomas have an underlying genetic association (such as von Hippel Lindau syndrome, multiple endocrine neoplasia type 2, and neurofibromatosis type 1), so all patients with paraganglioma should be offered genetic screening [3].

A recent structured review described 94 cases of ACTH-producing pheochromocytoma or paraganglioma—with only 20 being paragangliomas—highlighting the rarity of this diagnosis [1]. Comparatively distinct was the degree of hypercortisolism. Our patient's morning serum cortisol was 5-fold above the reference range and 24-hour urine cortisol was 165-fold above the reference range, compared to the structured review in which median morning serum cortisol was 3.3-fold above the reference range and 24-hour urine cortisol was 21.3-fold above the reference range [1]. This case also demonstrates a use of osilodrostat—chosen for its potent and rapid control of hypercortisolism—which normalized cortisol levels in just more than 4 weeks, allowing the patient to proceed with surgery. Of the 94 cases reviewed, none used osilodrostat [1].

We describe a newer approach to the perioperative management of ACTH-dependent hypercortisolism by using osilodrostat [4, 5]. Osilodrostat works by inhibiting 11β-hydroxylase, which converts 11-deoxycortisol to cortisol; this medication received Food and Drug Administration approval in 2020 for management of CD in those who are not surgical candidates or failed surgical intervention [6]. This case demonstrates that osilodrostat may be similarly used to manage Cushing syndrome (CS), especially when alternative therapies are contraindicated. A recently published review of trials using osilodrostat for CD and CS found it to be effective with a favorable safety profile [7]. Close laboratory and symptomatic monitoring are essential to maintain appropriate therapeutic levels. Twenty-four–hour urine free cortisol monitoring is optimal but logistically challenging. For this patient, 24-hour urine and random serum cortisol levels both were monitored and correlated well. When she developed adrenal insufficiency, dose reduction plus replacement hydrocortisone allowed safe surgical intervention. Postoperative corticosteroid replacement with slow taper is needed given prolonged excess cortisol.

Prophylaxis and treatment for complications of excess cortisol and catecholamines were important to this patient's care. Guidelines for hypertension in CS suggest initially prioritizing angiotensin blockade for hypertension management [8]. Our patient demonstrated improved response to α and β blockade, suggesting a substantial role of metanephrine excess in her hypertension. Patients with notably elevated cortisol levels, classified as severe CS, are at increased risk for venous thromboembolism [9]. The Endocrine Society suggests considering thromboprophylaxis in those with a urine free cortisol level more than 5-fold above normal (in addition to standard risk factors) [10]. Additionally, PCP prophylaxis should be considered given immunocompromise from prolonged steroid exposure [10].

## Learning Points

- Paragangliomas most classically secrete catecholamines, but alternative cosecreted hormones should be considered as suggested by the clinical picture.
- Osilodrostat may be an option to achieve cortisol suppression in hypercortisolism even in ectopic CS prior to procedural intervention.
- Hypercortisolism, as a risk factor for hypercoagulability and immunosuppression, should prompt consideration for thrombosis and infection prophylaxis.

## Data Availability

Original data generated and analyzed for this case report are included in this published article.

## Abbreviations

ACTH: adrenocorticotropin
bpm: beats per minute
CD: Cushing disease
CS: Cushing syndrome
CT: computed tomography
DHEA-S: dehydroepiandrosterone sulfate
FDG-PET: fluorodeoxyglucose–positron emission tomography
IV: intravenous
PCP: *Pneumocystis jirovecii* pneumonia
